# Extracting Multiple Worries From Breast Cancer Patient Blogs Using Multilabel Classification With the Natural Language Processing Model Bidirectional Encoder Representations From Transformers: Infodemiology Study of Blogs

**DOI:** 10.2196/37840

**Published:** 2022-06-03

**Authors:** Tomomi Watanabe, Shuntaro Yada, Eiji Aramaki, Hiroshi Yajima, Hayato Kizaki, Satoko Hori

**Affiliations:** 1 Division of Drug Informatics Keio University Faculty of Pharmacy Tokyo Japan; 2 Nara Institute of Science and Technology Nara Japan; 3 Mediaid Corporation Tokyo Japan

**Keywords:** breast neoplasm, cancer, natural language processing, NLP, artificial intelligence, model, machine learning, content analysis, text mining, sentiment analysis, oncology, quality of life, social media, social support, breast cancer, BERT model, peer support, blog post, patient data

## Abstract

**Background:**

Patients with breast cancer have a variety of worries and need multifaceted information support. Their accumulated posts on social media contain rich descriptions of their daily worries concerning issues such as treatment, family, and finances. It is important to identify these issues to help patients with breast cancer to resolve their worries and obtain reliable information.

**Objective:**

This study aimed to extract and classify multiple worries from text generated by patients with breast cancer using Bidirectional Encoder Representations From Transformers (BERT), a context-aware natural language processing model.

**Methods:**

A total of 2272 blog posts by patients with breast cancer in Japan were collected. Five worry labels, “treatment,” “physical,” “psychological,” “work/financial,” and “family/friends,” were defined and assigned to each post. Multiple labels were allowed. To assess the label criteria, 50 blog posts were randomly selected and annotated by two researchers with medical knowledge. After the interannotator agreement had been assessed by means of Cohen kappa, one researcher annotated all the blogs. A multilabel classifier that simultaneously predicts five worries in a text was developed using BERT. This classifier was fine-tuned by using the posts as input and adding a classification layer to the pretrained BERT. The performance was evaluated for precision using the average of 5-fold cross-validation results.

**Results:**

Among the blog posts, 477 included “treatment,” 1138 included “physical,” 673 included “psychological,” 312 included “work/financial,” and 283 included “family/friends.” The interannotator agreement values were 0.67 for “treatment,” 0.76 for “physical,” 0.56 for “psychological,” 0.73 for “work/financial,” and 0.73 for “family/friends,” indicating a high degree of agreement. Among all blog posts, 544 contained no label, 892 contained one label, and 836 contained multiple labels. It was found that the worries varied from user to user, and the worries posted by the same user changed over time. The model performed well, though prediction performance differed for each label. The values of precision were 0.59 for “treatment,” 0.82 for “physical,” 0.64 for “psychological,” 0.67 for “work/financial,” and 0.58 for “family/friends.” The higher the interannotator agreement and the greater the number of posts, the higher the precision tended to be.

**Conclusions:**

This study showed that the BERT model can extract multiple worries from text generated from patients with breast cancer. This is the first application of a multilabel classifier using the BERT model to extract multiple worries from patient-generated text. The results will be helpful to identify breast cancer patients’ worries and give them timely social support.

## Introduction

Breast cancer is the most diagnosed female cancer worldwide, and treatment can last for 5 to 10 years, making this a familiar disease that women will live with for a long time [1-3]. Patients with breast cancer have multiple worries about treatment, family, finances, and so on, and these worries change over time. Although support for them is provided by medical professionals, patients’ worries are sometimes overlooked in clinical settings [4].

Currently, many patients use social media as a source of medical information [5]. Patient-generated text such as posts and comments are accumulated on the internet and contain a wealth of information about patients’ experiences and daily worries. It may be possible to use this information to help patients solve their problems and improve their quality of life. However, the substantial amount of text and the variable reliability of information on social media make it difficult for patients to get the accurate information they seek [6]. This large amount of social media data has become a new source of medical information and a target for natural language processing (NLP) [7,8].

Document classification by NLP can be used to extract information from text. This technique is useful for automatically identifying worries from patient-generated text and helping patients with breast cancer obtain appropriate information to resolve their worries. Although there are many NLP studies on portals for patients with breast cancer, most of them are content analyses that objectively analyze the contents of media. Although content analysis research can find multiple worries, the extracted worries cannot be defined. In contrast, document classification can set target worries and find them, but so far, there have been few document classification studies [9], and studies targeting worries are particularly rare. Therefore, it is necessary to create a document classification model that can automatically extract multiple worries from text generated from patients with breast cancer.

There has been much research on using NLP to extract topics and worries from patient-generated text automatically. Many studies used rule-based, bag-of-words, and topic models such as latent Dirichlet allocation (LDA) [10-12], and there remains room for improvement in extracting worries from the variously expressed patient descriptions in these models. These models have particular difficulty in dealing with context, but context can be used by deep-learning methods such as long short-term memory (LSTM) and Bidirectional Encoder Representations From Transformers (BERT), which has proved to be state of the art in several NLP tasks [13]. While there have been studies of patient-generated text using BERT to extract adverse drug effects [14,15], few studies have been conducted on text describing multiple worries that patients often have at the same time. There are some previous reports in which sentiment classification of patient-generated text was conducted using LSTM [16]. However, these only apply one label to one document and do not address multiple worries within a single document.

The purpose of this study was to develop a multilabel classification model using BERT to automatically extract multifaceted worries from text generated by patients with breast cancer.

## Methods

### Data Set

**Figure 1:**
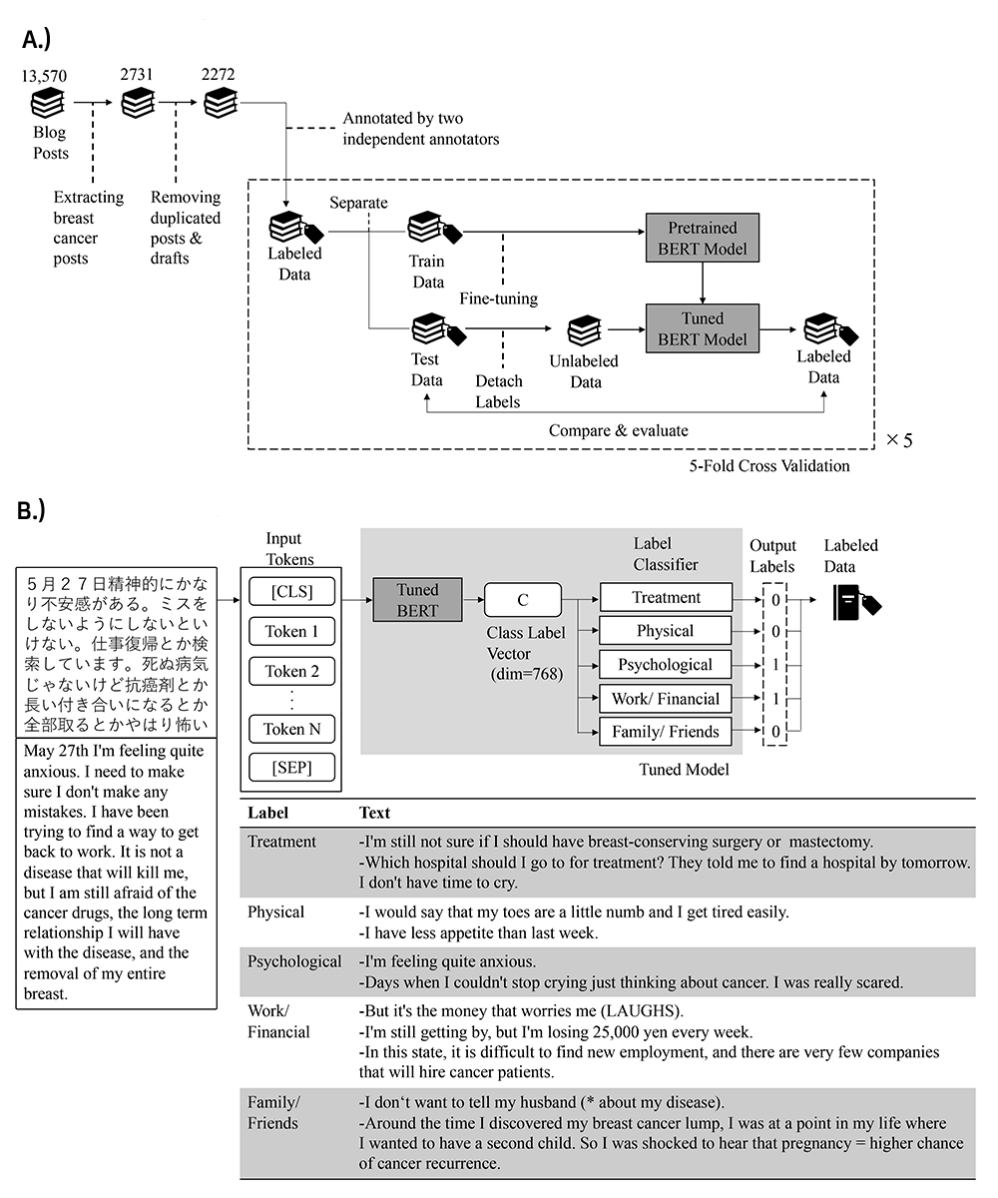
Overview of data processing and model function. (A) Data selection criteria and model training and testing process; (B) post label prediction model functions and outputs. *In Japanese sentences, the object is sometimes omitted, so the presumed object was judged from the context and added in parentheses. BERT: Bidirectional Encoder Representations From Transformers.

In this study, blog articles on Life Palette [17], one of the internet patient communities in Japan, were used. All the articles were written in Japanese. The data source consists of 13,570 posts written by 289 users from March 2008 to November 2014. A total of 2272 breast cancer posts were extracted as a data set, excluding drafts and duplicates (Figure 1).

### Ethical Approval

This study was approved by the ethics committee of the Keio University Faculty of Pharmacy (approval No 191218-2, 190301-1). All procedures were performed in accordance with the Ethical Guidelines for Medical and Health Research Involving Human Subjects (settled by the Ministry of Education, Culture, Sports, Science and Technology and the Ministry of Health, Labour and Welfare in Japan) and the Declaration of Helsinki and its later amendments. Consent to use the data from Life Palette for research purposes was obtained at the time of user registration. In this study, all data were analyzed anonymously and informed consent for this research was waived due to the retrospective observational design of the study.

### Annotation

The annotation criteria were defined based on previous studies [18]. To assess the reliability of the annotation criteria, 50 blog posts were randomly selected from the data set and annotated by two researchers with medical knowledge (authors TW and SH). After assessment of interannotator agreement (IAA) by means of Cohen kappa, one researcher (TW) annotated all the blogs. Cohen kappa takes a value close to 1 if the annotators are in perfect agreement; less than 0 is *poor*, 0-0.2 is *slight*, 0.21-0.4 is *fair*, 0.41-0.6 is *moderate*, 0.61-0.8 is *substantial*, 0.81-1 is *almost perfect* [19].

Based on the “Shizuoka Classification” [20], which is a method for classifying the worries of patients with cancer in Japan, the following five labels were established: “treatment,” “physical,” “psychological,” “work/financial,” and “family/friends” (Table S1 in Multimedia Appendix 1). If a single blog post contains descriptions of multiple worries, multiple labels were allowed.

### Model Structure

In this study, a multilabel classifier was built from the annotated multilabel data set to deal with multiple descriptions of worries. To develop the classifier, BERT, a state-of-the-art NLP model that can take context into account, was used. BERT is trained via a two-step learning process. The first step is pretraining using a large amount of text data and the second step is fine-tuning the model from new data.

The model was built by fine-tuning the pretrained Japanese BERT model of the Inui and Suzuki Laboratory, Tohoku University [21] (BERT-base model; 12 layers, 768 dimensions of hidden states, and 12 attention heads, tokenizer: MeCab [22], external dictionary: mecab-ipadic-NEologd [23]) from the annotated multilabel data set. Due to the capability of the pretrained model, the input was limited to 512 words, starting from the beginning of the sentence.

**Figure 2:**
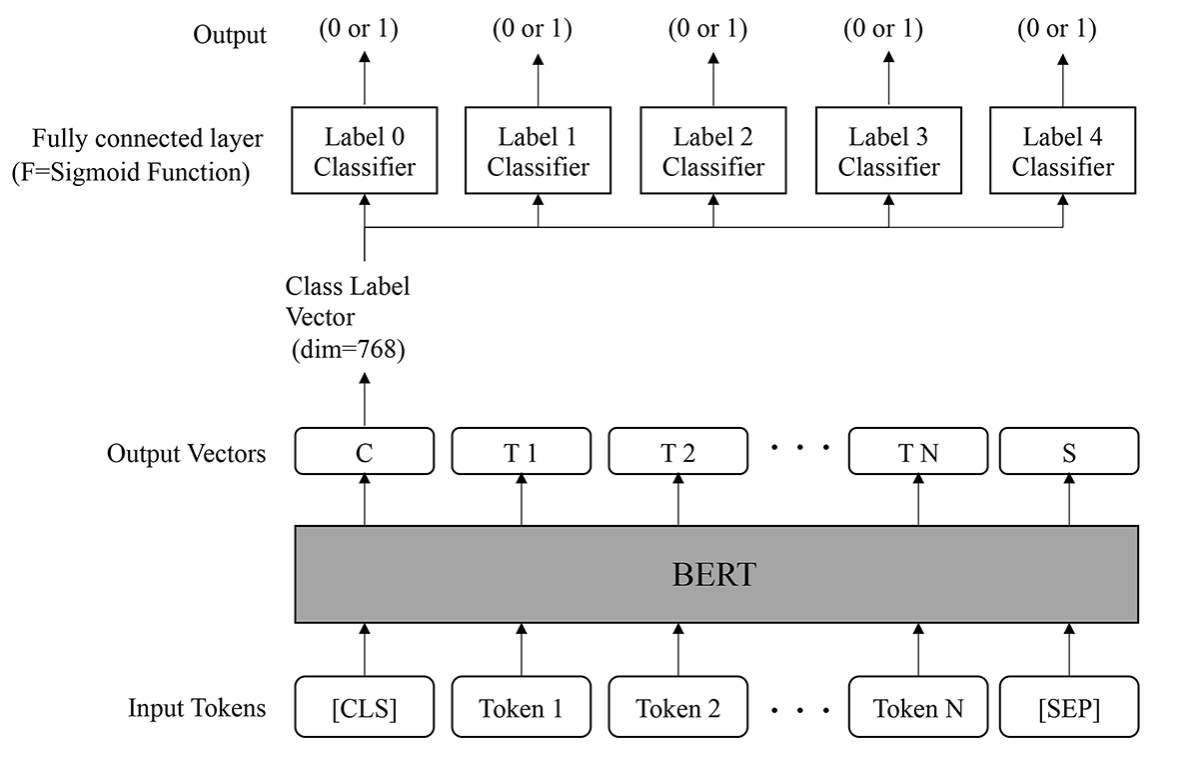
Model structure developed in this study. The input is the post sentence with [CLS] token and [SEP] token added at the beginning and at the end, respectively. The output is 0/1, corresponding to negative/positive of each label. BERT: Bidirectional Encoder Representations From Transformers; dim: dimension.

The [CLS] token and [SEP] token were added at the beginning of the sentence and at the end of the sentence, respectively. This was used as input to the BERT model. The model consists of a pretrained BERT and a fully connected layer, and the activation function was a sigmoid function that outputs five labeled positive/negative results. The model was built with reference to the previous study [24]. The input to the fully connected layer was the vector corresponding to the [CLS] token in the output vector of the pretrained BERT. The hyperparameters that could be adjusted prior to training were defined as follows. The loss function was cross-entropy, batch size was 16, five epochs were run, early stopping was not set, and all parameters were fine-tuned, including the pretrained BERT from Adam with a learning rate of 1e-5 (Figure 2).

In the BERT model, it is possible to incorporate a self-attention method that allows indicating which part of the output text has been paid attention to. Visualizing the attentions can be useful in interpreting the results of “black box” machine learning models. Therefore, in this study, the attention parts of each blog post were visualized and used as a reference for interpreting the labeling results.

### Task and Metrics

A multilabel task was performed to classify five labels simultaneously. The performance was evaluated in terms of precision, *F* score, and exact match accuracy, which indicates the percentage of correct predictions for all labels. As a way to use the research, we envision the construction of an information provision system tailored to each patient’s problems. Therefore, we focused on precision so as not to provide unmatched information and inadvertently impose a burden on patients with breast cancer. The data set was divided into training data and test data in a ratio of 4:1, and the model was evaluated using the average of 5-fold cross-validation results to confirm its robustness.

Moreover, to examine the effect of the upper limit of the number of input words on the model performance, the performance for blog posts with over 512 words, that for all posts, and that for posts with 512 words or less were compared.

## Results

### Data Set Analysis

The mean number of words per blog post in the data set was 464.9, the median was 357, and the maximum was 6746. The number of documents with more than 512 words was 723 (31.8% of all blog posts; Figure S1 in Multimedia Appendix 1).

### Annotation

The IAA values were the highest for “physical” and the lowest for “psychological” (Table 1). This time, the labels except for “psychological” showed a high degree of agreement with IAA values higher than 0.61, corresponding to “substantial” precision. The complete label agreement rate that indicates all the label-matched blog posts was 0.40.

The number of blog posts was highest for “physical” and lowest for “family/friends” (Table 1). The number of labels per blog post was the highest for single label posts and the lowest for posts with all five labels. Articles with no labels at all amounted to 544 (23.9%), and articles with a single label and multiple labels amounted to 892 (39.3%) and 836 (36.8%), respectively (Table 2). In addition, it was found that there were differences in worries among users, and the worries expressed by the same user changed over time (Figure S2 in Multimedia Appendix 1).

**Table 1:** The IAA^a^ values and the number of posts for the five labels (N=2272).

Label	IAA^b^	Posts, n
Treatment	0.67	477
Physical	0.76	1138
Psychological	0.56	673
Work/financial	0.73	312
Family/friends	0.73	283

^a^IAA: interannotator agreement.

^b^Annotation agreement was evaluated using Cohen kappa.

**Table 2:** The number of labels per blog post (N=2272).

Number of labels	Posts, n (%)
0	544 (23.9)
1	892 (39.3)
2	578 (25.4)
3	199 (8.8)
4	57 (2.5)
5	2 (0.1)

### Model

The precision was 0.59 for “treatment,” 0.82 for “physical,” 0.64 for “psychological,” 0.67 for “work/financial,” and 0.58 for “family/friends.” Both the precision and the *F* score were highest for “physical” (Table 3). The exact match accuracy was 0.44.

The performances of posts with more than 512 words and posts with 512 words or less are presented in Multimedia Appendix 1.

**Table 3:** Performance of the model.

Label	Accuracy (SD)	Precision (SD)	Recall (SD)	*F* score (SD)
Treatment	0.81 (0.01)	0.59 (0.09)	0.39 (0.15)	0.44 (0.09)
Physical	0.81 (0.01)	0.82 (0.02)	0.80 (0.02)	0.81 (0.01)
Psychological	0.77 (0.03)	0.64 (0.04)	0.54 (0.08)	0.58 (0.04)
Work/financial	0.88 (0.02)	0.67 (0.10)	0.28 (0.05)	0.38 (0.03)
Family/friends	0.88 (0.02)	0.58 (0.11)	0.33 (0.07)	0.41 (0.07)
Macro average	0.83 (0.01)	0.66 (0.04)	0.47 (0.05)	0.52 (0.03)

## Discussion

### Principal Findings

This is the first report of a multilabel classifier using the BERT model to extract multiple types of worries in patient-generated text, and our results indicate that BERT is effective for this purpose.

### Comparison With Prior Work

Our model can extract multiple worries from a single post. There have been some NLP studies that have dealt with multiple worries in patient-generated text [18,25]. However, these studies used a multi-class classification that allows only one label per document and could not find multiple worries contained in a single document. Similar to this study, there was a previous study on classifying blog sentences with worry descriptions [18]. However, the previous study dealt with binary classification and short text, while our study dealt with multilabel classification and long text. Furthermore, our study outperformed the previous one in *F* score. Some studies have used a multilabel classifier of patient-generated messages based on the viewpoint of medical professionals [26,27]. In contrast, a noteworthy feature of this study was the classification of patient-generated text from the viewpoint of patients.

### Strength of the Model

A multilabel classifier may be useful for patients with breast cancer because they may have multiple worries and the nature of their worries may change over time. This study has demonstrated that documents with multiple worries can be handled using BERT. As another approach, a lot of content analysis research has been done using topic models such as LDA for unsupervised learning [10]. LDA is a model that extracts multiple topics in a single document that would be suitable for handling a wide range of patient worries. However, this model is often used for content analysis rather than document classification, which ultimately requires manual interpretation of topics. An advantage of our model is that it automatically outputs the presence or absence of worries based on the input of sentences, so it does not require a final human judgment and can present the results quickly. Thus, our context-aware model is expected to be efficient for dealing with texts generated by patients with breast cancer that contain multiple worries and long descriptions because it extracts worries by paying attention to descriptions based on the human senses (Figure S3 in Multimedia Appendix 1).

### Features of the Data Set

The reliability of the data set was inferred from the annotation results: the IAA was above 0.61, which was “substantial” for all labels except “psychological,” indicating a high degree of agreement. The “psychological” label tended to be judged differently among researchers, compared with the other labels. However, it is considered that the data set was reliable enough as training data because the IAA values exceeded 0.41, which indicates “moderate” reliability. In the data set of posts written by patients with breast cancer, more than one worry was actually described in about 40% of the posts (Table 2), and it was confirmed that the worries described by the same user changed over time (Figure S1 in Multimedia Appendix 1), which was in agreement with previous studies. These results suggest that the data set was suitable for development of a multilabel classifier.

### Error Analysis

To evaluate the reliability of the model, error analysis was conducted. Many of the false-positive cases were descriptions of changes in “physical,” which had the highest precision, and dealt with conditions that were not covered by the annotation guidelines. They were similar to the “physical” descriptions, such as postoperative recovery, chest discomfort before diagnosis, and changes in physical condition that seemed unrelated to cancer (eg, “I was surprised that I could lift my arms more than before surgery!” “One day, I was surprised at the size of the difference between my left and right breasts,” or “I drank a little wine and sake and felt dizzy”). Although there is still room for improvement in the performance of this model in discriminating between “presence of distress” and “presence of distress caused by breast cancer,” this model will be useful in supporting patients with breast cancer because we were able to extract descriptions of “physical changes that cause distress” in patients with breast cancer.

### Limitations

First, the BERT model used in this study has great strength in recognizing context, but the upper limit of the number of input words is 512. Although there was concern that the performance might deteriorate with posts having more than 512 input words, it was found that there was almost no difference between the performance only for posts with more than 512 input words and that for all posts. On the other hand, the performance for posts with 512 input words or less was slightly inferior to that for all posts. Based on these results, it was considered that truncation after 512 input words had little effect on the model performance, whereas the lack of information due to a small number of input words had a greater effect in this analysis. This suggests that blog posts containing a larger number of input words than the upper limit would not degrade model performance (Table 2 and Table S2 in Multimedia Appendix 1).

Second, the small number of blog posts for each label in our data set is also the limitation of this study. Our model was built from the data set containing descriptions of five worry types. The prediction performance of the model was different for each label, and the higher the IAA and the greater the number of posts, the higher the precision and the *F* score tended to be. This suggests that the IAA and the number of posts are important factors in constructing the classifier. This problem can be overcome by increasing the number of blog posts for each label.

Third, the patients’ blogs used in this study were written in Japanese. It is important to develop a classification model in Japanese, but the lack of applicability to multiple languages may be a limitation.

### Future Directions

Our findings could lead to the development of better patient support systems and methods that can respond to temporal and interindividual changes in worries. Our methodology also facilitates the identification of worries and may promote the sharing of problems among patients. Furthermore, in the future, by combining sentiment analysis with our model, it might be possible to enrich the interpretation of the findings and deepen the understanding of how breast cancer patients’ worries influence their emotions. Although this study focused only on worries about breast cancer, there are many common worries that are not specific for breast cancer, and it is expected that the model could be extended to other disease areas.

### Conclusion

In conclusion, this study showed that the BERT model can extract multiple worries, such as “treatment,” “physical,” “psychological,” “work/financial,” and “family/friends,” from text generated by patients with breast cancer. This is the first study to deal with multiple patient worries using BERT and demonstrates the usefulness of NLP techniques in dealing with patient-generated text. The results will be helpful to identify breast cancer patients’ worries and give them timely social support.

## Appendix

Multimedia Appendix 1 Supplementary material.

## Abbreviations

BERT: Bidirectional Encoder Representations From Transformers
IAA: interannotator agreement
LDA: latent Dirichlet allocation
LSTM: long short-term memory
NLP: natural language processing

## Data Availability

The data consisting of blog articles in the study are available from Mediaid Corporation upon reasonable request.
