# Supplementary material for: Extracting Multiple Worries From Breast Cancer Patient Blogs Using Multilabel Classification With the Natural Language Processing Model Bidirectional Encoder Representations From Transformers: Infodemiology Study of Blogs
Source: JMIR Cancer. 2022 Jun 3;8(2):e37840. doi: 10.2196/37840 (PMC9206207; doi:10.2196/37840)
Supplement: Multimedia Appendix 1 [file cancer_v8i2e37840_app1.docx]

Table S1.　Annotation guideline

| Label | Detail |
| --- | --- |
| Treatment | - Being jealous of healthy visitors and pregnant women - I want to stay out of the hospital, but I can't - Worries about postponing treatment - Anxiety about the end of treatment - Concerns about:   Outpatient, hospitalization, discharge, and transfer   - Hospital response, facilities, and relationships with medical personnel - Notification, informed consent, and second opinions - Diagnosis and treatment - Medical treatment - Not being able to take a clinical trial - Clinical trial selection - Response of the local hospital after discharge - Postponing treatment |
| Physical | - Concerns about cancer symptoms, side effects of medication, and after-effects of treatment   (Label any side effects if they occur.)  (Also label hair loss, loss of fertility, etc.)   - Fever, malaise, etc. - Abnormal values - Insomnia - Physical pain due to medical treatment |
| Psychological | - Vague anxiety about cancer - Anxiety about recurrence - Depression, despair, shock, sadness, fear |
| Work/financial | - Worries about high medical costs, work, and coworkers - The burden of cancer on work |
| Family/friends | - Worries about family, loved ones, children, and local community - I take it out on my family. - Having a hard time being treated as a sick person by others - Feeling guilty about them - Worries about housework - Worries about pregnancy and partner |


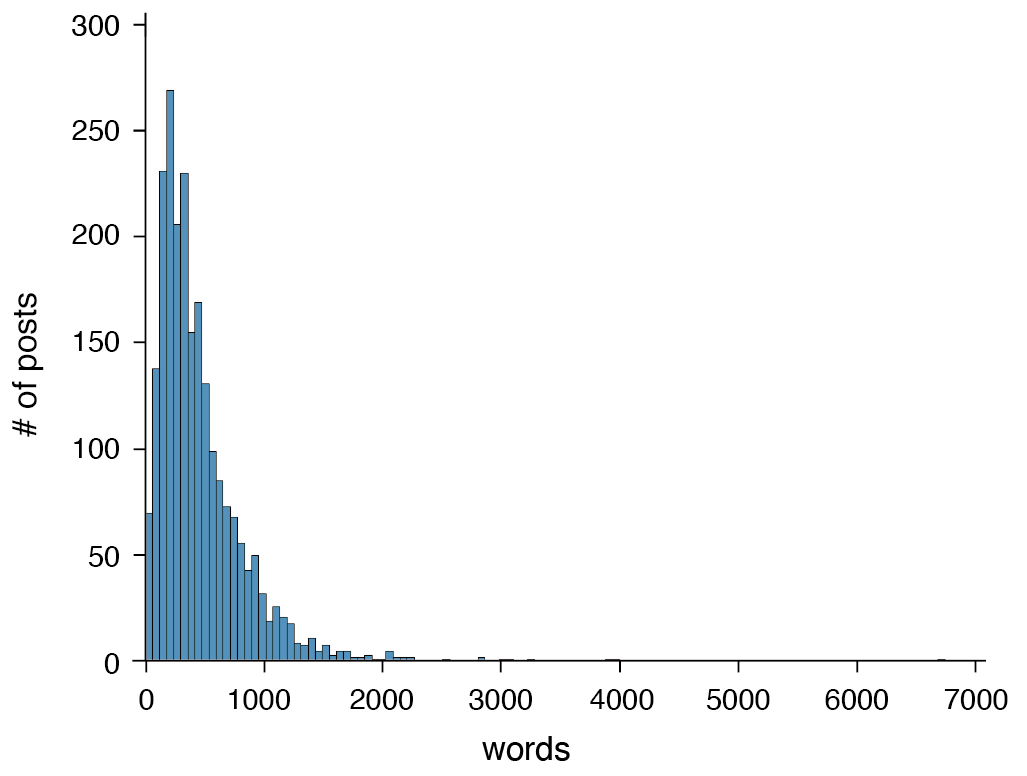


Figure S1. Histogram of the number of words per post (n = 2,272 posts)

The mean number of words per blog post in the dataset was 464.9, the median was 357.0, and the maximum was 6,746.

#
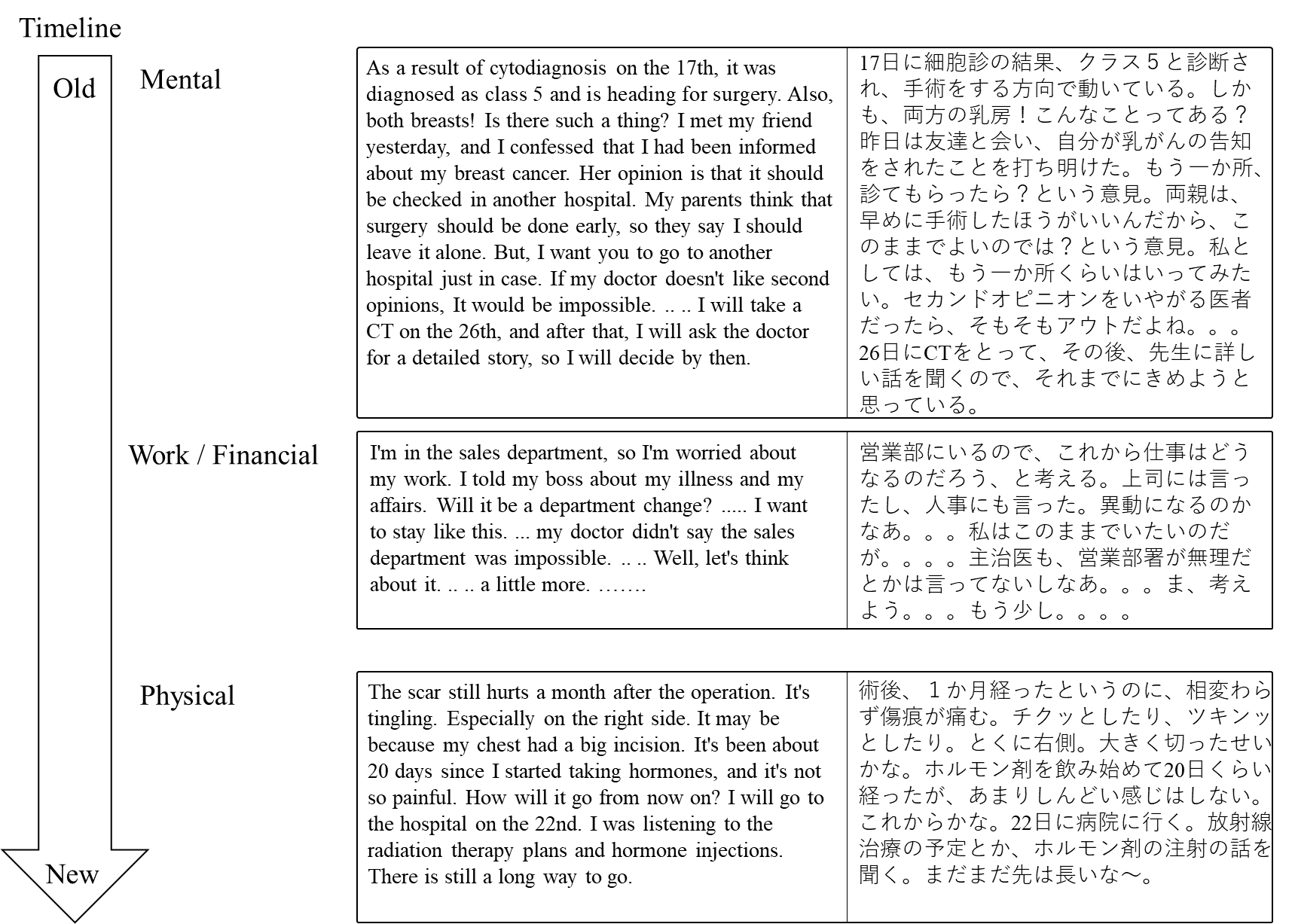
Figure S2. Changes in problems over time

An example of how one user's concerns change

Table S2. Model performance for the posts over 512 words.

| Label | Accuracy (SD) | Precision (SD) | Recall (SD) | F-score (SD) |
| --- | --- | --- | --- | --- |
| Treatment | 0.70 (0.01) | 0.63 (0.09) | 0.53 (0.17) | 0.55 (0.09) |
| Physical | 0.79 (0.01) | 0.82 (0.03) | 0.80 (0.05) | 0.81 (0.02) |
| Psychological | 0.67 (0.01) | 0.62 (0.07) | 0.55 (0.09) | 0.58 (0.03) |
| Work/financial | 0.83 (0.01) | 0.69 (0.14) | 0.26 (0.07) | 0.36 (0.06) |
| Family/friends | 0.81 (0.01) | 0.60 (0.10) | 0.36 (0.09) | 0.44 (0.08) |
| Macro average | 0.76 (0.01) | 0.67 (0.05) | 0.50 (0.06) | 0.55 (0.03) |

Table S3. Model performance for the posts 512 words or less.

| Label | Accuracy (SD) | Precision (SD) | Recall (SD) | F-score (SD) |
| --- | --- | --- | --- | --- |
| Treatment | 0.86 (0.02) | 0.49 (0.12) | 0.23 (0.15) | 0.28 (0.10) |
| Physical | 0.82 (0.01) | 0.81 (0.03) | 0.80 (0.03) | 0.80 (0.01) |
| Psychological | 0.82 (0.03) | 0.65 (0.06) | 0.54 (0.07) | 0.59 (0.05) |
| Work/financial | 0.90 (0.02) | 0.66 (0.09) | 0.29 (0.06) | 0.39 (0.06) |
| Family/friends | 0.92 (0.02) | 0.54 (0.11) | 0.28 (0.06) | 0.37 (0.07) |
| Macro average | 0.86 (0.01) | 0.63 (0.04) | 0.43 (0.04) | 0.49 (0.03) |

Figure S3.　Examples of blog post attention

Examples of true-positive are shown below. For these posts, the model correctly predicted the labels. The redder the color, the more attention is received. The label names following the post number show all the labels attached to that post.

*(cont’d)*Figure S3.　Examples of blog post attention

Examples of true-positive are shown below. For these posts, the model correctly predicted the labels. The redder the color, the more attention is received. The label names following the post number show all the labels attached to that post.
